# Supplementary material for: The causal role of circulating inflammatory markers in osteoporosis: a bidirectional Mendelian randomized study
Source: Front Immunol. 2024 Jul 18;15:1412298. doi: 10.3389/fimmu.2024.1412298 (PMC11291241; doi:10.3389/fimmu.2024.1412298)
Supplement: Supplementary file 1 [file DataSheet_1.zip › supplementary material/CIM-OSTEOPOROSIS /GCST90274827.csv_leave_one_out.pdf]

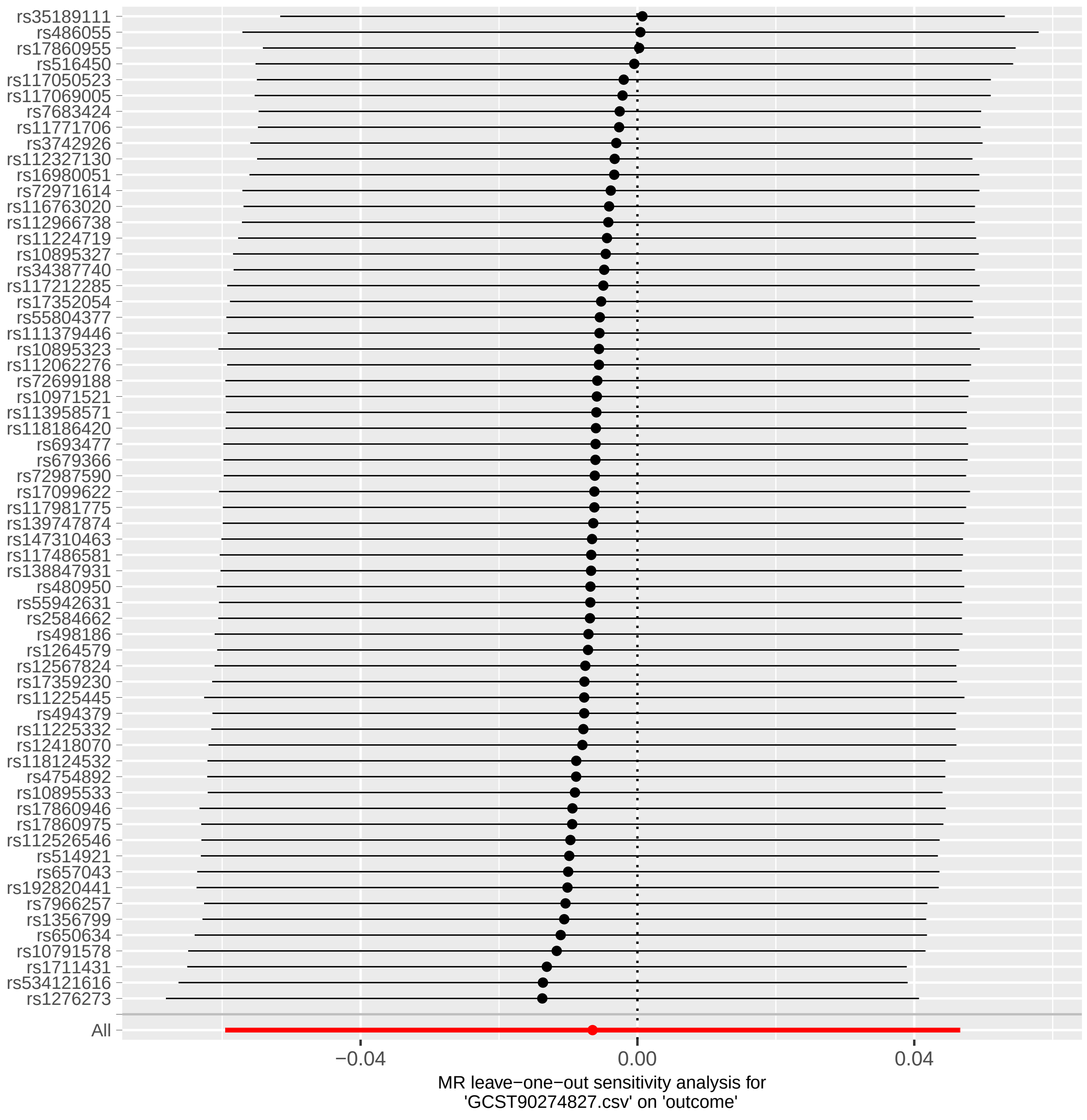
rs35189111
rs486055
rs17860955
rs516450
rs117050523
rs117069005
rs7683424
rs11771706
rs3742926
rs112327130
rs16980051
rs72971614
rs116763020
rs112966738
rs11224719
rs10895327
rs34387740
rs117212285
rs17352054
rs55804377
rs111379446
rs10895323
rs112062276
rs72699188
rs10971521
rs113958571
rs118186420
rs693477
rs679366
rs72987590
rs17099622
rs117981775
rs139747874
rs147310463
rs117486581
rs138847931
rs480950
rs55942631
rs2584662
rs498186
rs1264579
rs12567824
rs17359230
rs11225445
rs494379
rs11225332
rs12418070
rs118124532
rs4754892
rs10895533
rs17860946
rs17860975
rs112526546
rs514921
rs657043
rs192820441
rs7966257
rs1356799
rs650634
rs10791578
rs1711431
rs534121616
rs1276273
All
−0.04
0.00
0.04
MR leave−one−out sensitivity analysis for
'GCST90274827.csv' on 'outcome'
